# Cough expired volume and airflow rates during sequential induced cough

**DOI:** 10.3389/fphys.2013.00167

**Published:** 2013-07-05

**Authors:** Karen W. Hegland, Michelle S. Troche, Paul W. Davenport

**Affiliations:** ^1^Speech Language and Hearing Sciences, University of FloridaGainesville, FL, USA; ^2^Physiologic Sciences, University of FloridaGainesville, FL, USA

**Keywords:** cough, reflex cough, airway protection, sequential cough, cough effectiveness

## Abstract

Cough effectiveness is determined by a combination of volume of air expired and maximum expiratory airflow rate. Studies of cough sensitivity identify cough thresholds based on at least 2 or 5-cough re-accelerations to a stimulus, however, to date no study has examined the interplay between the distribution of cough expired air and cough airflow rates for these induced sequential coughs. The goal of this study was to investigate the relationship between reflex cough re-accelerations, cough airflow and cough inspired and expired volume. Twenty adults (18–40 years, four men) volunteered for study participation, and were outfitted with a facemask in-line with a pneumotachograph and a one-way valve for capsaicin delivery on inspiration. Cough inspired and expired volume (Liters of air) as well as airflow parameters (peak expiratory flow rates L/s) were measured for each cough response. Results demonstrate significant linear relationships between cough expired volume, flow rates, and the total number of coughs produced. Thus, as the number of coughs in an epoch increase, the mechanical effectiveness of coughs within the epoch may decrease according to peak expiratory flow rates and cough expired volume, particularly for coughs comprised of more than 3 re-accelerations.

## Introduction

The function of cough is to protect the airway, both by expelling material that has erroneously entered the airway during deglutition, or by removing endogenously produced material. To achieve effective airway clearance the rate and shearing forces associated with cough expiratory airflow must be sufficient to expel material. To this end, a single cough consists of three phases of airflow: the inspiratory phase, compression phase, and the expiratory phase. Depending on the type of cough being produced (e.g., voluntary vs. irritant-induced) and associated instructions (e.g., “take a big breath in and cough” vs. “cough when you need to”), the inspiratory phase may vary in terms of both duration and volume of inspired air, leading to small but significant differences in the expiratory phase of cough (Smith et al., 2012). Following the inspiratory phase, subglottal pressure increases during the compression phase. Vocal fold opening and concurrent expiratory muscle contraction mark the onset of the expiratory phase, leading to a rapid rise in the rate of expiratory airflow, and subsequent peak and sustained flows that carry material out of the airway (Harris and Lawson, 1968). The compression phase and expiratory phase may continue to alternate, producing a cough sequence, or epoch, consisting of multiple cough re-accelerations associated with the initial inspiration (Davenport et al., 2007). This organization (one inspiration followed by multiple alternating compression and expiratory phases) is the pattern associated with most irritant-induced or reflexive coughs.

There is a predictable relationship between cough peak flow and the number of cough re-accelerations produced within a cough epoch (Davenport et al., 2007; Smith et al., 2012). For example, a single voluntary cough or the first cough in a sequential cough epoch has higher peak flow rates vs. subsequent cough re-accelerations (Smith et al., 2012). This relationship is necessary given the interplay between the location of material to be removed from the airway (e.g., trachea vs. mainstem bronchi vs. smaller airways) and dynamic compression that occurs with cough production. The decrease in cross-sectional area resulting from dynamic compression increases the linear velocity of airflow at the site of compression, thereby greatly increasing the effectiveness of the cough (Ross et al., 1955; Macklem, 1974). The location within the airway where dynamic compression occurs is related to the equal pressure point, which is in turn related to lung volume. As a result, it is suggested that coughs are most effective at removing material from larger airways at lung volumes above functional residual capacity. As lung volume decreases and the equal pressure point moves more distally, secretions are moved from the smaller airways to the larger ones, where they can then be expelled upon subsequent inspiration and cough bouts (Macklem, 1974). Thus, multiple cough re-accelerations in an epoch, with associated decreasing lung volume, are imperative for removal of material from the lower airways.

Harris and Lawson (1968) showed that for three voluntary cough re-accelerations, the first, second and third coughs (Cr1, Cr2, and Cr3) comprised a mean of 53.2, 28.5, and 18.8% cough expired air, respectively. The first cough (Cr1) had peak airflow velocities that ranged between 1.6 and 2.1 times the magnitude of the second cough (Cr2), and from 3.2 to 4.8 times those of the third cough (Cr3), thus demonstrating a decrementing relationship among successive serial cough re-accelerations. Because the peak flows accounted for only 21–25% of the “scrubbing action” (a measure of cough effectiveness), the authors conclude that the total expired air and sustained airflow are more important than peak airflow alone in assessing the effectiveness of a cough (Harris and Lawson, 1968). Thus, these results in combination with the conclusions of Macklem (1974) suggest that for cough function to be effective it is necessary to: (1) achieve high peak *and* sustained airflow, resulting in adequate air expired during a cough, and (2) produce subsequent coughs at lower and lower lung volumes in order to move material from the smaller airways toward larger airways, which can then be expelled during subsequent inspirations.

Studies of cough sensitivity identify cough thresholds based on at least 2 or 5-cough re-accelerations to a stimulus (Dicpinigaitis and Alva, 2005). In their 2007 study, Vovk and colleagues (2007) examined type of cough response (i.e., the number of cough re-accelerations) in terms of the muscle activation, airflow, and duration of cough re-accelerations. The results revealed highest expiratory airflows and cough volume accelerations for the first cough (Cr1) in the series. However, the relationship between inspired air, total expired air volume and peak flow rate has not been investigated for multiple re-accelerations during reflexive cough epochs as it has been for voluntary cough.

There are known differences between voluntary and induced reflexive cough in terms of the functional organization of motor activity and its relationship to cough airflow, however, the actual cough airflows have been found to be similar between voluntary and reflex cough (Lasserson et al., 2006). The goal of this study was to investigate the relationship between reflex cough re-accelerations, cough airflow and cough inspired and expired volume in order to determine whether the airflow distributions are similar to those found previously for sequential voluntary cough (Harris and Lawson, 1968). It was first hypothesized that there would be a direct linear relationship between cough inspired and expired volumes. Second, it was hypothesized that there would be a direct linear relationship between cough expired volume and peak expiratory flow rate. Third, it was hypothesized that inspiratory and expiratory volumes would increase with increasing number of coughs in an epoch. Lastly, it was hypothesized that significant differences would exist for the volume of air expired and peak expiratory flow rate according to the place of a cough re-acceleration within an epoch (i.e., 1st vs. 4th re-acceleration), and total number of cough re-accelerations in an epoch.

## Methods

Twenty adults (18–40 years, four men) volunteered for study participation and gave verbal and written informed consent. All participants denied a history of chronic cough, current or chronic respiratory disease, asthma, smoking within the last 5 years, neurological disorder, head and/or neck cancer, and dysphagia (disordered swallowing). Pulmonary function testing and oral motor examination confirmed forced expiratory volume in 1 s/forced vital capacity of >75% predicted and integrity of cranial nerve function, respectively. The institutional review board at the University of Florida approved the study.

### Equipment

Participants were outfitted with a facemask covering the nose and mouth. The facemask was coupled to a pneumotachograph, differential pressure transducer (Validyne MP45) and had a side delivery port with a one-way inspiratory valve for nebulizer connection. The nebulizer was a DeVilbiss T-piece (DeVilbiss Healthcare) connected to a dosimeter (Koko Dosimeter) that delivered aerosolized solution during inspiration with a delivery duration of 2 s. Participants were administered single inhalations of aerosolized 200 μ M capsaicin dissolved in a vehicle solution consisting of 80% physiological saline, 10% Tween 20, and 10% ethanol. The concentration of 200 μm was chosen based on the study by Vovk and colleagues (2007) that found 200 μm to be a suprathreshold concentration for eliciting the C5 response in healthy participants. The airflow signal was recorded to a desktop computer (Dell Optiplex 745) via PowerLab Data Acquisition System (ADInstruments).

Participants were seated comfortably for an initial 30 s of quiet breathing in order to acclimate to the facemask, and provide a measure of baseline tidal volume (V_T_). Participants were then given the instruction “cough if you need to” prior to capsaicin delivery. The capsaicin solution was automatically administered upon detection of an inspired breath, and three trials were completed with a minimum of 1 min between each trial. Participants were provided water to drink as needed between trials.

## Data analysis and statistics

**Figure 1:**
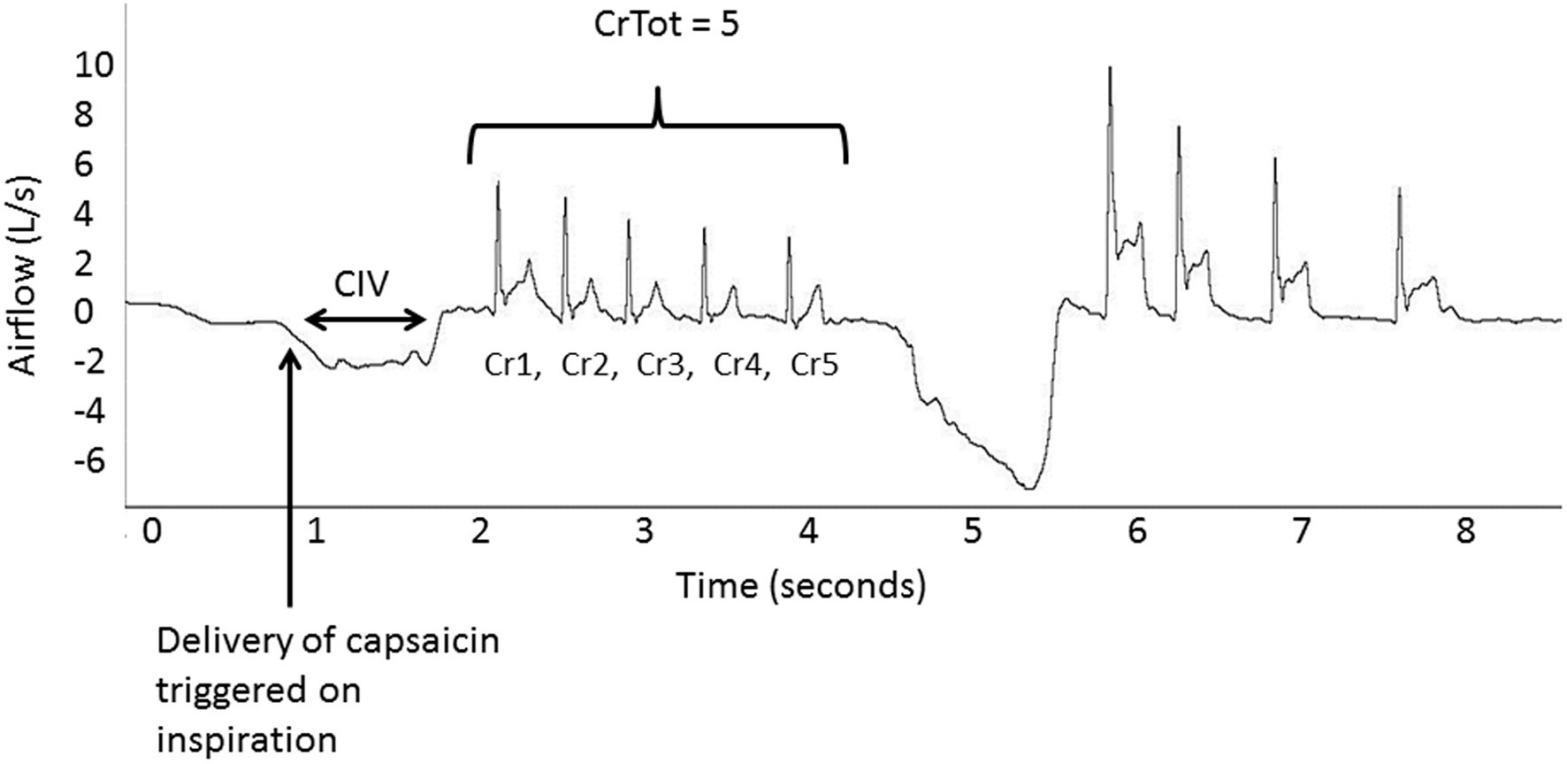
**Airflow recorded with delivery of the capsaicin stimulus indicated with the arrow**. CIV is cough inspired volume; Cr is cough re-acceleration; CrTot is total re-accelerations within an epoch.

For the purpose of this study, a cough (Cr1) is defined as inspiration followed by an expiratory effort against a closed glottis (compression phase), followed by glottal opening and rapid expiratory airflow. Associated re-accelerations (CrNs) following the initial cough (Cr1), also include the compression phase, glottal opening and rapid expiratory airflow, but are not preceded by inspiration (Figure 1). A cough epoch was defined as the Cr1 and all subsequent CrNs (Figure 1). Participants were not told how many coughs or cough re-accelerations to produce, so there was variability in the total number of cough re-accelerations (CrTot) within an epoch, ranging from 2- cough expulsive responses (C2); to 7-cough expulsive responses (C7). Each first-cough acceleration (Cr1) and all associated re-accelerations (Cr*2* – Cr*7*) were analyzed (Figure 1). The following measures were computed from all coughs within an epoch:

Cough inspiratory volume (CIV; L)
Peak expiratory flow rate (PEFR; L/s)
Total expired volume (CEV_T_; the sum of CEV from all CrNs in the epoch)
%CEV (the percent of CEV_T_ expired per Cr)

These measures for Cr1 and all associated CrNs were calculated across the three cough trials per participant. The raw data was used for groupwise analyses because of the within and between participant variability in number of CrNs produced per trial.

Bivariate correlation analyses using Pearson's r were used to determine the relationship between cough inspired volume, total expired volume (CEV_T_) and CrTot, and between PEFR and %CEV. A two-way multivariate analysis of variance (MANOVA) was used to test the hypothesis that significant differences would exist for PEFR and %CEV, according to both CrN and CrTot. *Post-hoc* testing was performed using Tukey's HSD. Significance for each model was set at *p* < 0.05, with adjustments made for multiple comparisons.

## Results

**Figure 2:**
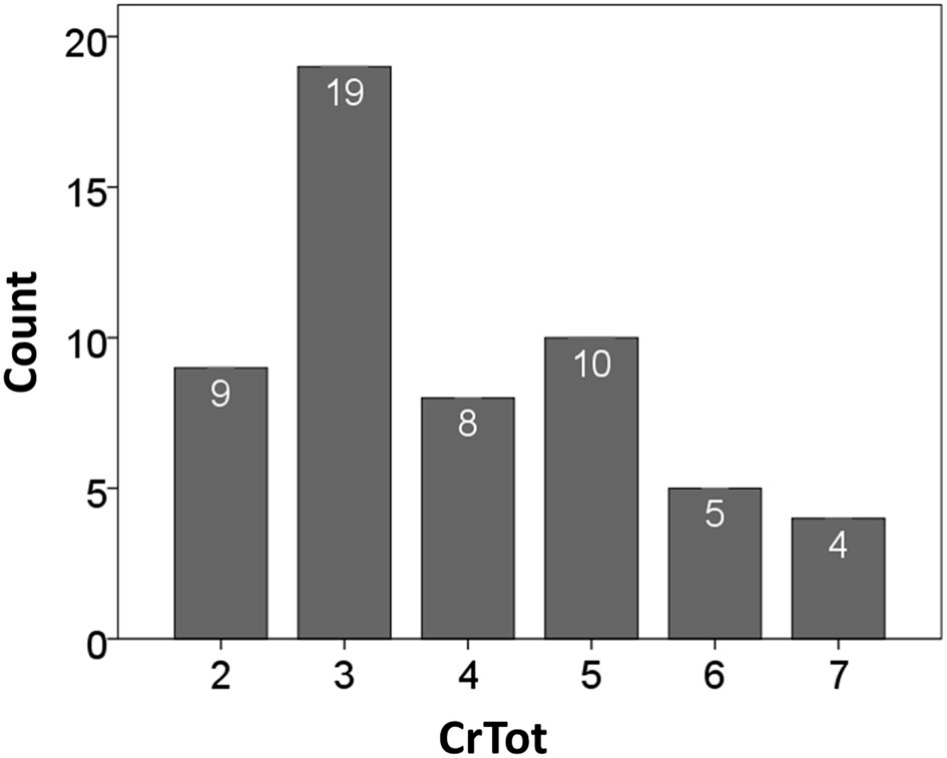
**Distribution of cough epochs according to CrTot**.

The overall distribution of CrTot across all participant responses is in Figure 2. Three coughs (C3) was the most common response to the capsaicin stimulus. Results of the bivariate correlation analyses for CIV, CEV_T_, and Cr Tot are in Table 1. Results of the correlation analysis between %CEV and PEFR revealed a moderate positive correlation (*r* = 0.578; *df* = 55; *p* < 0.001).

**Table 1:** **Results of the bivariate correlation analyses between CEV_T_, CIV, and CrTot**.

	**CrTot**	**CIV**	**CEV**_**T**_
CrTot	–	−0.367^*^	−0.262
CIV	−0.367^*^	–	0.685^**^
CEV_T_	−0.262	0.685^**^	–

Note: CEV_T_ is total cough expired volume; CIV is cough inspired volume; CrTot is total coughs in an epoch.

* p = 0.007;

** p < 0.001.

**Figure 3:**
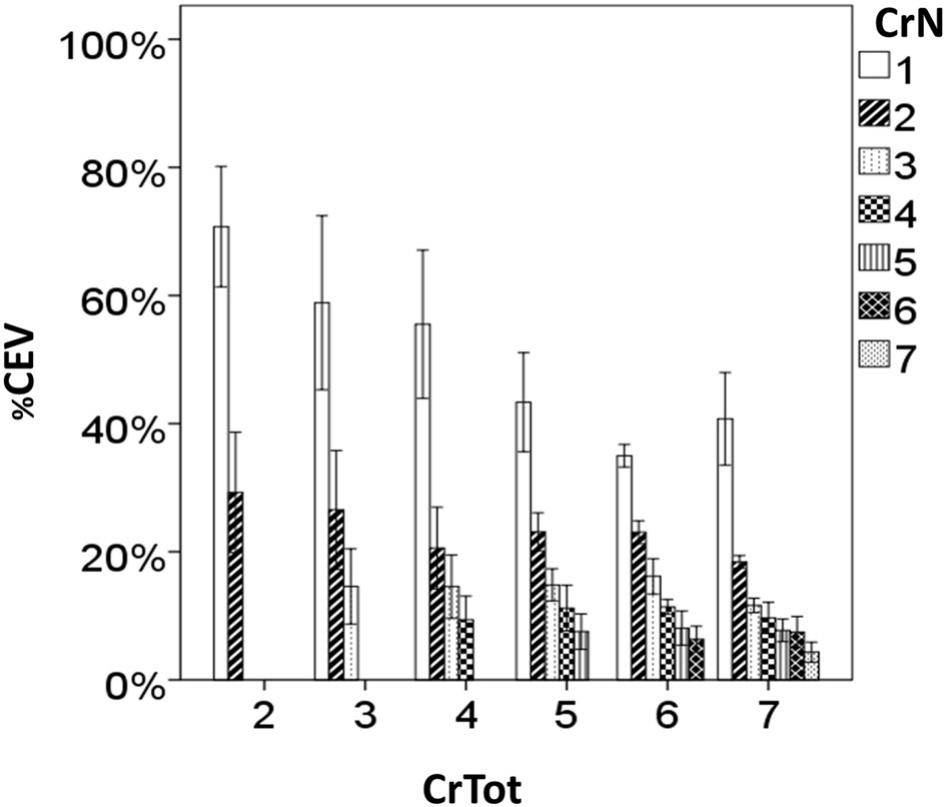
**Percent cough expired volume (%CEV) for each cough re-acceleration number (Cr*N*), categorized according to the total number of cough re-accelerations within the epoch (CrTot)**.

**Figure 4:**
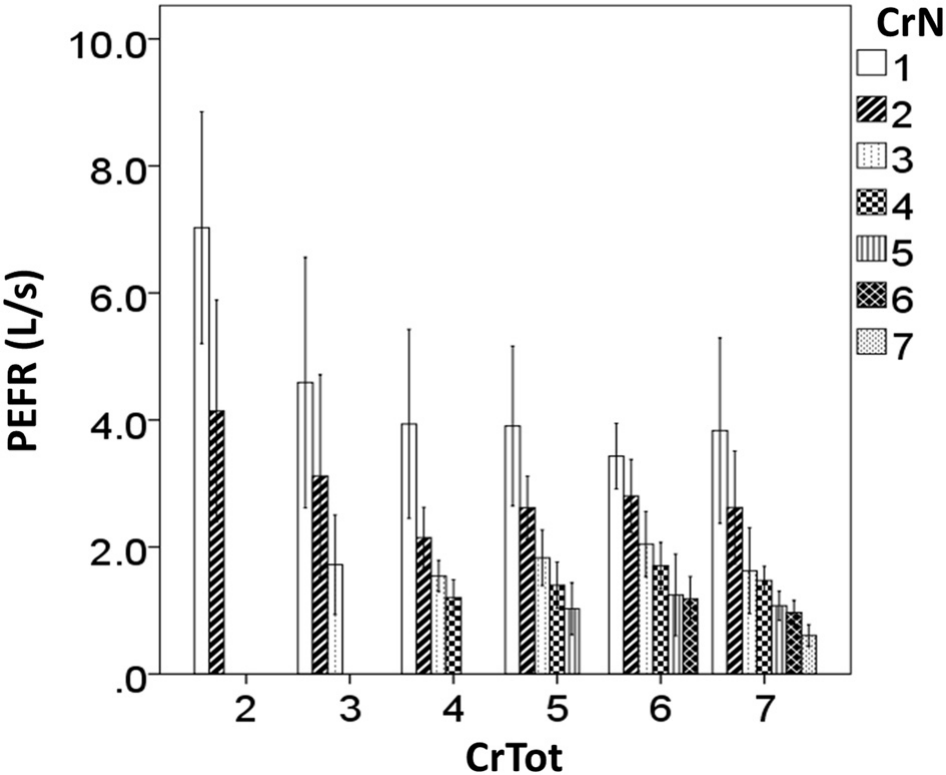
**Peak expiratory flow rate (PEFR) for each cough re-acceleration number (Cr*N*), categorized according to the total number of cough re-accelerations within the epoch (CrTot)**.

Results of the two-way MANOVA revealed significant overall two-way interaction effect for CrN and CrTot (Table 2). The interaction effect was significant for %CEV (Table 2) but not for PEFR. There were significant main effects of CrN and CrTot on PEFR (Table 2). Further illustration of these findings is in Figures 3, 4.

**Table 2:** **Multivariate (MANOVA) and Univariate (ANOVA) analyses of variance *F* ratios for CrN × CrTot effects for PEFR and %CEV**.

	**MANOVA**	**ANOVA**	
		**PEFR**	**%CEV**
	CrN: *F*_(12, 398)_	CrN: *F*_(6)_	CrN: *F*_(5)_
	CrTot: *F*_(10, 398)_	CrTot: *F*_(6)_	CrTot: *F*_(5)_
	CrN × CrTot:	CrN × CrTot:	CrN × CrTot:
	*F*_(30, 398)_	*F*_(15)_	*F*_(15)_
CrN (Cough number)	37.084^**^	25.135^**^	110.401^**^
CrTot (Total coughs)	11.061^**^	15.914^**^	17.418^**^
CrN × CrTot	2.133^*^	0.620	4.131^**^

Only those measures with significant *p*-values are included.

Note: MANOVA F ratio is Wilks' Lambda approximation of F. PEFR is peak expiratory flow rate; %CEV is percent cough expired volume.

* p = 0.001;

** p < 0.001.

## Discussion

The goal of this study was to determine the relationship between reflex cough re-accelerations, cough airflow, cough inspired volume and cough expired volume. Overall, the data supported the a priori hypotheses, revealing significant positive correlations between inspired and expired volumes, and significant differences in cough expiratory airflow measures depending upon the place and total number of cough re-accelerations in an epoch.

**Figure 5:**
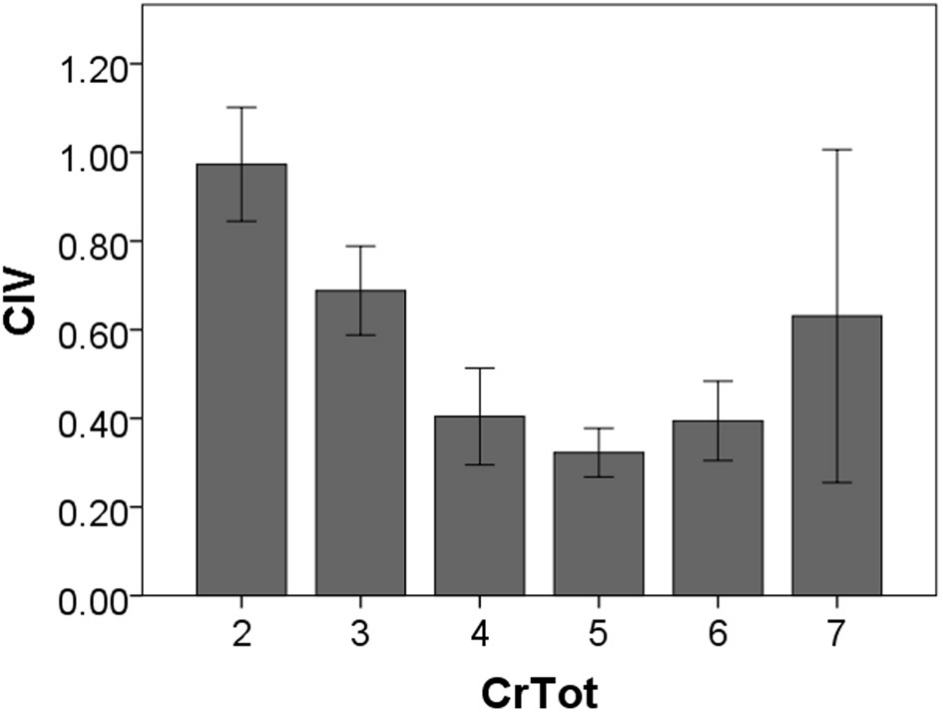
**Cough inspired volume according to the total number of cough re-accelerations within the epoch (CrTot)**.

Cough inspired and expired volumes increase in a direct, linear fashion. This corroborates findings of Smith and colleagues (2012) relating amount of air inhaled prior to voluntary cough production. Specifically, Smith et al. (2012) found that for single voluntary coughs, higher operating volumes (defined as the amount of air in the lungs, expressed as a percent of vital capacity, at cough initiation) were directly related to increased volume change during the cough expiratory phase (cough expired volume). Interestingly, the correlation results show that there is not a significant relationship between CrTot and CEV_T_, which is surprising given the expectation that as total coughs increase so would total expired volume. *Post-hoc* examination of the cough expired volume of only the first cough in the epoch reveals a moderate negative correlation with CrTot, showing that as CrTot increases the total air expired from the first cough (Cr1) decreases. These results, along with those of Smith and colleagues, suggest that air volume inspired prior to the cough relates best to the total air volume expired during either a single cough (Smith et al., 2012) or an the first cough in a sequential cough epoch. Interestingly, our results show that the total number of coughs in an epoch is negatively correlated with CIV, indicating that as the number of coughs within an epoch increases, CIV actually decreases, as can be seen in Figure 5.

These findings may relate to the perceived intensity of the cough-inducing stimulus. Davenport and colleagues (2009) have shown that when urge-to-cough, a measure of perceived stimulus intensity, increases the total number of coughs increases. In this study, the stimulus magnitude (200 μM capsaicin) was consistent across participants, suggesting *perceived* magnitude was an important component of the cough response. It may be that when greater stimulus intensity is perceived, the inspiratory phase terminates earlier, resulting in reduced total inspired volume. At the same time, the great perceived intensity may lead to more coughing, and increased CrTot, leading to a distribution of expired volume that is specifically decreases for Cr1, allowing for redistribution of that air for additional CrNs in the epoch. Studying the urge-to-cough as it relates to CIV and CEV will be an important component of future study on irritant-induced cough.

Further analysis of the expired volume distribution across coughs within an epoch reveals that for irritant-induced sequential cough epochs, the expired volume for Cr1 ranged between 40 and 70% total air expired in the epoch. This percentage varied according to the total number of coughs in the epoch, illustrating the significant impact of CrTot on the *distribution* of expired air, but not the total air expired. The %CEV for the second cough in the epoch (Cr2) also decreased with increasing CrTot, averaging about 30% for a CrTot of 2, and 20% for a CrTot of 7. However, for CrNs (place in an epoch) of 3 and greater the effect of CrTot disappears, with a relatively consistent %CEV for Cr3 − Cr7 in longer cough epochs. This shows that the re-distribution of expired air for increasing CrTots relies heavily on changes in Cr1 %CEV, to a lesser extent Cr2 %CEV, and not on subsequent cough re-accelerations.

In addition to decreasing %CEV, peak expiratory flow rate (PEFR) also decreased according to CrTot, and according to place within the epoch (CrN). This is in-line with recent findings of Smith and colleagues (2012), who reported a direct relationship between volume change (measure similar to %CEV used in the current study) and PEFR for the first, middle, and last cough re-accelerations within a voluntary cough “peal” (epoch). *Post-hoc* analysis shows that PEFR is always highest for Cr1, across all CrTot categories; it decreases significantly with increasing CrTot. A similar effect is seen for Cr2, however, the effect disappears for Cr3 in the remaining CrTot categories. This is similar to the pattern seen with %CEV. Together, these results demonstrate increasing number of coughs beyond approximately 3–4 in an epoch does not enhance cough effectiveness, and may have a detrimental effect whereby PEFR and %CEV actually decrease for Cr1 with cough epochs comprised of more than 3 coughs.

Examination of the mean data for CIV and CEV_T_ reveals that cough expired volume is between 2 and 2.5 times greater than inspired volume. Thus, more air is expired during a cough epoch of any length than is inspired; this indicates lung volumes below resting expiratory level (REL) are routinely utilized for irritant-induced cough. One consequence of cough expulsive efforts occurring in these relatively lower lung volumes is that dynamic compression occurring at low lung volumes decreases the cross-sectional area of the airway, and subsequently increases the linear velocity of airflow. This in turn moves material from the smaller airways toward larger airways, which can then be effectively expelled during subsequent inspirations (Ross et al., 1955; Macklem, 1974). The relatively consistent inspired and expired volumes across CrTot categories likely relates to this property, allowing for removal of material from smaller airways to assist in maintaining pulmonary health.

In summary, percent cough expired volume (%CEV) and peak expiratory flow rate (PEFR) are inversely related to total coughs within the epoch. As the number of coughs in an epoch increase, the mechanical effectiveness of coughs within the epoch may decrease, particularly for coughs comprised of more than 3 re-accelerations. Regardless of the total number of coughs in the epoch, the mechanical effectiveness as judged by %CEV and PEFR is always greatest for the first cough, and the effectiveness of subsequent coughs is relatively decreased. This may be related to the perception of stimulus magnitude that seems to be a driver of not only how many total coughs are produced (Davenport et al., 2009), but potentially inspired and expired air volume characteristics of the coughs. Perhaps the “ideal” cough pattern for effectively clearing material from the lower airways would consist of multiple 3-cough epochs, where the highest peak flows are achieved in combination with maintaining cough expired volume to remove material from smaller airways at those lower lung volumes. Ongoing and future research will determine the sequential cough epoch pattern in patient populations, perceived magnitude by measuring urge-to-cough, and how these parameters relate overall airway protection.

### Conflict of interest statement

The authors declare that the research was conducted in the absence of any commercial or financial relationships that could be construed as a potential conflict of interest.
